# Inclusiveness of the Concept of Mental Disorder and Differences in Help-Seeking Between Asian and White Americans

**DOI:** 10.3389/fpsyg.2021.699750

**Published:** 2021-07-30

**Authors:** Jesse S. Y. Tse, Nick Haslam

**Affiliations:** School of Psychological Sciences, University of Melbourne, Melbourne, VIC, Australia

**Keywords:** mental disorder, help-seeking, cultural differences, stigma, concept breadth, Asian American, White American

## Abstract

Ethnic and racial group differences in help-seeking are a barrier to the effective and equitable delivery of mental health services. Asian American populations demonstrate relatively low levels of help-seeking. Explanations for this effect typically point to elevated levels of stigma in these populations. An alternative explanation is that low help-seeking might also reflect holding a relatively circumscribed concept of mental disorder. Individuals and groups with less inclusive concepts of disorder may be less likely to identify problems as appropriate for mental health treatment. This study aimed to test whether group differences in the breadth of the mental disorder concept account for group differences in help-seeking attitudes. A sample of 212 American participants (102 Asian Americans and 110 White Americans) were assessed on personal stigma, help-seeking attitudes, and mental disorder concept breadth. Mediation analyses examined whether stigma and concept breadth mediated group differences in attitudes. Compared to White Americans, Asian Americans reported higher levels of stigma and narrower concepts of mental disorder, both of which were associated with less positive help-seeking attitudes. Stigma and concept breadth both partially mediated the group difference in attitudes. Theoretical and practical implications for mental health promotion and culturally sensitive clinical practices are explored.

## Introduction

Many people who might benefit from mental health treatment do not receive it (Corrigan et al., 2014). The underutilization of services has been identified as a barrier to improved public mental health, and promoting appropriate help-seeking is an essential step to overcoming it (Boonstra et al., 2012; Anderson et al., 2013). Understanding why people do not seek available help is therefore a pressing concern.

Help-seeking refers to any initiatives people take to engage with care (Kovandžić et al., 2011). Many conceptual models have been proposed to clarify its processes and determinants (e.g., Latané and Darley, 1970; Keith-Lucas, 1972; Goldberg and Huxley, 2012). Featherstone and Broadhurst (2003) argued that all models follow a three-stage structure: recognizing a psychological problem (stage 1), deciding to seek help (stage 2), and actively seeking help (stage 3). Help-seeking behavior, therefore, depends on people’s pre-existing beliefs and attitudes about mental disorders and their treatment (Loya et al., 2010). Help-seeking attitudes are a particularly important antecedent (Kraus, 1995; Eisenberg et al., 2009; Masuda and Boone, 2011), greatly increasing people’s likelihood of seeking professional help (Mojtabai et al., 2002). Therefore, studying the predictors of help-seeking attitudes is a research priority.

Demographic factors, such as gender, consistently predict help-seeking attitudes (e.g., Rickwood and Braithwaite, 1994; Oliver et al., 2005; Mackenzie et al., 2006) and race and ethnicity are also clearly implicated. In the United States, for example, most minority groups underutilize mental health services relative to White individuals (Sheu and Sedlacek, 2004; Kearney et al., 2005; Abe-Kim et al., 2007; Kam et al., 2019). In particular, evidence has accumulated over several decades that Asian Americans, as a diverse group with different languages and cultural beliefs, engage in mental health services at relatively low rates (Abe-Kim et al., 2007; Lee et al., 2011; Cheng et al., 2013) and hold less positive help-seeking attitudes than other Americans (Masuda et al., 2009; Masuda and Boone, 2011). Studies indicate that Asian Americans are two to five times less likely to seek help than their White peers (Garland et al., 2005; Eisenberg et al., 2007).

Stigma is often invoked to explain people’s reluctance to seek help for mental health problems. It is defined as a social process that results in people being disqualified from full social acceptance (Goffman, 2009). It is inevitably grounded in cultural norms and values (Corrigan, 2000), and as a result, levels of stigma vary between cultural groups (Narrow et al., 2000; Corrigan, 2004). Ethnic and racial group differences in levels of stigma – i.e., people’s endorsement of negative stereotypes of those experiencing mental disorders (e.g., perceived dangerousness) and desired social distance from them (Griffiths et al., 2004) – are now well established (Leong and Lau, 2001; Griffiths et al., 2006; Eisenberg et al., 2009). Asian Americans, in particular, report higher levels of stigma than other groups (Eisenberg et al., 2009; Cheng et al., 2013), including White individuals (Griffiths et al., 2006; Rao et al., 2007; Jimenez et al., 2013), a difference possibly rooted in concerns about bringing shame to the family (Gee et al., 2020). Higher levels of stigma among Asian Americans also bear on their help-seeking attitudes (Cooper et al., 2003; Eisenberg et al., 2009; Masuda and Boone, 2011), most likely obstructing the second, “decision-making” stage of the help-seeking process (Eisenberg et al., 2009; Schomerus et al., 2009; Clement et al., 2015). Consistent with this possibility, Loya et al. (2010) found that differences in stigma mediated differences in help-seeking attitudes between White and South Asian American college students. Although Asian Americans consistently revealed higher level of stigma than their White counterparts, this difference, and the factors that account for it, may vary substantially for people from the many different ethnicities grouped together in that broad category. For instance, Chinese Americans were found to underutilize mental health services due to the perceived shame rather than the disbelief in Western health services (Yang et al., 2008), whereas cultural mistrust is one of the factors that relates to the negative help-seeking attitudes among Filipino Americans (David, 2010).

Other than stigma, how different groups conceptualize mental disorder may also help to explain differences in help-seeking attitudes. One possible group difference in conceptualization that has yet to be examined is the inclusiveness of the concept of mental disorder. Referred to here as “concept breadth,” the inclusiveness of this concept is the range of psychological phenomena that are identified as disordered by a person or group. A narrower concept includes a smaller range, setting a higher threshold for identifying behavior or experience as disordered. As a construct, concept breadth is distinct from stigma because it involves a belief about the semantic range of the concept of mental disorder rather than an evaluative attitude to people with mental disorders.

The idea that concept breadth might be psychologically important was raised by Haslam (2016), who argued that harm-related concepts, such as “mental disorder,” have tended to broaden their lay and professional meanings in recent decades, a shift he dubbed “concept creep.” Similar concerns have been raised earlier in the psychiatric domain by writers criticizing diagnostic inflation and the pathologization of normal experience (e.g., Horwitz and Wakefield, 2007; Frances, 2013). Haslam proposed that concepts may creep vertically, expanding downward to encompass less severe phenomena, or horizontally, expanding to incorporate new kinds of phenomena. For example, loosening the diagnostic criteria for autism to encompass milder variants represent vertical creep, and referring to gambling as a behavioral addiction represents the horizontal creep of the concept of “addiction.” Because the concept of mental disorder is intrinsically fuzzy, historically fluid, and culturally shaped, people may vary substantially in the breadth of their concepts.

Many studies have explored cultural differences in understandings of disorder and cultural idioms of distress (e.g., Kohrt et al., 2014). For example, a study by Chentsova-Dutton and Ryder (2020) compared different cultural models of normalcy and deviancy, illustrating cultural variations in beliefs about what constitutes mental health and ill health. However, empirical studies that have established cross-cultural variation in the breadth of mental disorder concepts are scarce. Giosan et al. (2001) presented a set of 68 (translated) vignettes describing behaviors that might represent mental disorders – many corresponding to the *Diagnostic and Statistical Manual of Mental Disorders* (fourth ed.; *DSM-IV*; American Psychiatric Association, 1994) – to participants from Brazil, Romania, and the United States and asked whether the vignettes exemplified their local disorder concept. Americans had the broadest concept of mental disorder, followed by Romanians and Brazilians. A subsequent study by Glovsky and Haslam (2003) further showed the cultural dimension of differences in concept breadth, demonstrating that more acculturated Brazilian immigrants in the United States held more inclusive concepts of disorder. These findings raise the likelihood that Asian Americans, even those who are relatively acculturated, may hold narrower concepts of mental disorder than White Americans. However, to date, there has been no systematic investigation of differences in concept breadth between these groups in the United States.

Just as previous research has pointed to stigma as an influence on help-seeking attitudes, so may concept breadth be another plausible influence. A person holding a narrow concept of mental disorder should be less likely than one holding an inclusive concept to identify an experience or behavior as disordered, and thus less likely to recognize it as a problem requiring professional intervention, which is the first stage in the help-seeking process (Featherstone and Broadhurst, 2003). Such a person should tend to conceptualize mental disorder as a relatively severe and uncommon phenomenon – perhaps relying on lay conceptions of madness or insanity – relative to people whose concept is more expansive and whose threshold for identifying mental disorder is lower. Consequently, people holding narrow concepts of disorder should be less positively disposed than others to seek help for psychological problems, and especially for problems at the less severe end of the psychopathological spectrum.

On the basis of this reasoning, we anticipated that in addition to holding more stigmatizing perceptions of mental disorder than White Americans on average, Asian Americans might also hold narrower concepts of disorder. These narrower concepts might partially account for Asian Americans’ somewhat less positive attitude to help-seeking. To evaluate these predictions and test this novel psychological factor that might be implicated in help-seeking, we conducted the first study of the role of concept breadth in cultural group differences in help-seeking attitudes. In addition to asking whether broader concepts of disorder are indeed associated with more positive attitudes to mental health help-seeking, we examined whether stigma and concept breadth mediate the group difference in these attitudes. The following hypotheses were tested.

*H*_1_: Asian Americans would have less positive help-seeking attitudes than White Americans.

*H*_2_: Asian Americans would hold more stigmatizing views of people with mental disorders than White Americans.

*H*_3_: Participants who had more stigma would have less positive help-seeking attitudes.

*H*_4_: Participants’ stigma toward people with mental disorders would mediate the group difference in help-seeking attitudes.

*H*_5_: Asian Americans would have narrower concepts of mental disorder than White Americans.

*H*_6_: Participants with narrower concepts of mental disorder would have less positive help-seeking attitudes.

*H*_7_: The breadth of participants’ concept of mental disorders would mediate the group difference in help-seeking attitudes.

## Materials and Methods

### Participants

Participants were recruited using Amazon’s Mechanical Turk (MTurk) service. To ensure data quality, participation was restricted to people located in the United States, with a ≥98% human intelligence task (HIT) approval rate and the number of approved HITs ≥1,000 (Goodman et al., 2013; Peer et al., 2014). A brief screening survey was created to recruit a balanced number of people from both groups of interest, with eligible participants invited to participate in the main survey.

The minimum target sample size to detect a medium effect with a power of 0.95 at an alpha level of 0.05 was determined to be 107 using the G*Power 3.1 program (Faul et al., 2009). More specific to mediation analysis using a bias-corrected bootstrap procedure, a sample size of 148 was required to detect a medium effect according to the review by Fritz and MacKinnon (2007). Oversampling was undertaken to mitigate the potential exclusion of data due to low compliance. Of the initial sample of 282, 70 participants (24.82%) were excluded for failing one or more of the following requirements: (1) an attention check (Oppenheimer et al., 2009; Goodman et al., 2013), (2) no straight-line response on any measure, and (3) a completion time less than 6 min (Zhang and Conrad, 2014). Statistical analysis was carried out on the final sample of 212 participants, aged between 19 and 70 (*M_age_* = 36.02, *SD* = 10.43), and including 105 women and 107 men.

### Measures

#### Group

Participants self-identified themselves with the question “With which ethnic group do you most identify?” with Asian and White being the two options.

#### Stigma

Two self-report scales were used to assess frequently researched facets of stigma. The perceived dangerousness of people with mental disorders was measured by the *Dangerousness Scale* (Link et al., 1987). Participants were asked to rate their endorsement of eight statements (e.g., *One important thing about mental patients is that you cannot tell what they will do from 1 min to the next*.) on a 7-point Likert scale (1 = *strongly disagree* to 7 = *strongly agree*). High scores indicate greater perceived dangerousness of people with mental disorders. Desire for social distance from people with mental disorders was assessed using the *Social Distance Scale* (Link et al., 1987). Participants rated their willingness to interact with a person with a mental disorder on a 4-point scale (0 = *definitely willing* to 3 = *definitely unwilling*) for seven scenarios of varying degrees of intimacy (e.g., How would you feel about renting a room in your home to someone with a mental disorder?). Higher scores represent greater desired social distance.

#### Concept Breadth

Since there is no published scale measuring the breadth of the concept of mental disorder, a new vignette measure was designed and tested in this study. Participants were asked to read 15 vignettes, each containing three to five sentences describing a person who might have a mental disorder. The vignettes were written to be purposefully ambiguous to detect differences between participants in the breadth of their concept of mental disorder. They covered a wide range of disorders from the *Diagnostic and Statistical Manual of Mental Disorders* (fifth ed.; *DSM-5*; American Psychiatric Association, 2013) and targeted disorders that reflected vertical creep (relaxation of diagnostic criteria or subthreshold cases of existing disorders) or horizontal concept creep (new disorders) based on the changes from *DSM-IV*. Examples were drawn from diverse domains of psychopathology (e.g., mood, anxiety, psychotic, neurodevelopmental, substance use, obsessive-compulsive, trauma-related, and neurocognitive disorders). After reading each vignette, participants responded to the question “*To what extent do you agree that the person in the description has a mental disorder?*” on a 6-point scale (1 = *strongly disagree* to 6 = *strongly agree*). Higher scores represent higher agreement that the condition described is a disorder. Inspection of the mean ratings of the 15 concept breadth vignettes and of the latent structure of responses to them was planned so as to select an ideal subset of vignettes for the final scale. This selection exercise was undertaken to ensure the final measure fulfilled its purpose by containing a psychometrically coherent sample of vignettes that contained ambiguous or marginal examples of mental disorder (i.e., ideally rated near the midpoint of the scale). Details of this refining process are presented in the Results section.

#### Help-Seeking Attitudes

These attitudes were assessed using the inventory of attitudes toward seeking mental health services (IASMHS; Mackenzie et al., 2004). This 24-item scale is a revised version of the popular Attitudes Toward Seeking Professional Psychological Help scale (ATSPPH; Fischer and Turner, 1970), which had been criticized for its validity limitations (Dadfar and Friedlander, 1982; Surgenor, 1985; Fischer and Farina, 1995). The IASMHS measures attitudes toward psychological counseling services *via* agreement on a 5-point Likert scale (0 = *disagree* to 4 = *agree*) with a series of statements (e.g., *If I believed I were having a mental breakdown, my first inclination would be to get professional attention*). One subscale, assessing “indifference to stigma,” was excluded due to its conceptual overlap with the stigma scales used in this study (Loya et al., 2010). Mackenzie et al. (2004) reported a high internal consistency of 0.86 for the IASMHS and a 3-week test-retest reliability of 0.73.

### Procedure and Design

This research project was approved by the Human Research Ethics Committee of the University of Melbourne. Participants were recruited from the MTurk platform and screened based on cultural group membership. Eligible participants gave consent and responded to the battery of questionnaires in the specific order of the concept breadth, dangerousness, social distance, and help-seeking scales. Participants were debriefed and paid for their participation.

### Statistical Analysis

To ensure the coherence of the new concept breadth measure, its items were examined using analysis of means and exploratory factor analysis (EFA). Scale reliability was assessed using the Guttman’s (1945) lambda-2 (*λ*_2_) coefficient. Correlation analysis tested *H*_1_-*H*_3_, *H*_5_, and *H*_6_. Mediation analyses were conducted using the SPSS PROCESS v2.1 (Hayes, 2013) to test for *H*_4_ and *H*_7_, following the recommendations of Hayes (2013) and Kane and Ashbaugh (2017). Accordingly, the significance and magnitude of effects were assessed using a bias-corrected bootstrap procedure (Hayes, 2009; Zhao et al., 2010; Rucker et al., 2011). As known correlates of help-seeking attitudes, age and gender were controlled in the mediation analyses (Sheu and Sedlacek, 2004; Oliver et al., 2005; Shea and Yeh, 2008; Rüsch et al., 2014). All statistical analyses were performed using the IBM SPSS Statistics Version 25.0.

## Results

### Preliminary Analysis

The 102 Asian Americans (*M_age_* = 32.57, *SD* = 7.93) were younger than the 110 White Americans (*M_age_* = 39.22, *SD* = 11.42), *t*(195.35) = −4.95, *p* < 0.001, 95% CI [−9.30, −4.00], and also more educated (*Mdn_(Asian)_* = 4, *Mdn_(White)_* = 4; *U* = 4672.50, *p* = 0.03, *r* = 0.15) and had higher annual income (*Mdn_(Asian)_* = 3, *Mdn_(White)_* = 2; *U* = 3686.50, *p* < 0.001, *r* = 0.32). There was no difference in gender composition, *χ^2^*(2; *N* = 212) = 3.21, *p* = 0.07.

### Refining the Concept Breadth Measure

As planned, EFA was conducted to ensure the unidimensionality of the measure. The scree plot, parallel test, and MAP test all suggested a two-factor solution. Due to severe multivariate non-normality and correlated constructs, the principal axis factoring extraction method was used with direct oblimin rotation (Fabrigar et al., 1999; Costello and Osborne, 2005; Williams et al., 2010). One vignette had cross-loadings on the two factors, nine loaded exclusively on the first factor and five loaded on the second factor. The five vignettes loading only on the second factor had the lowest mean mental disorder ratings of the 15 (range 1.81–2.98), indicating that they were insufficiently marginal, with very few participants judging the person in the description to have a mental disorder (i.e., rating ≥4). These five vignettes were therefore removed, so the refined measure represented the mean rating of the 10 vignettes loading on the first factor.

### Descriptive Statistics

The means, standard deviations, and reliability estimates of each measure for each subsample are summarized in Table 1. The overall Guttman’s (1945) lambda-2 reliability estimates for dangerousness, social distance, concept breadth, and help-seeking attitudes were 0.87, 0.90, 0.80, and 0.82, respectively. The concept breadth measure in the White American subsample had the lowest but still acceptable reliability (*λ*_2_ = 0.75).

**Table 1:** Descriptive statistics and reliability estimates of all measures.

Measures	Total (*n* = 212)		Asian (*n* = 102)		White (*n* = 110)	
	*Mean (SD)*	*λ*_2_	*Mean (SD)*	*λ*_2_	*Mean (SD)*	*λ*_2_
Dangerousness	3.73 (1.22)	0.87	4.01 (1.11)	0.83	3.47 (1.26)	0.89
Social distance	1.54 (0.68)	0.90	1.72 (0.65)	0.88	1.38 (0.67)	0.91
Concept breadth	3.78 (0.78)	0.80	3.66 (0.85)	0.82	3.89 (0.70)	0.75
Help-seeking attitudes	41.97 (9.71)	0.82	40.31 (9.33)	0.80	43.51 (9.84)	0.83

Correlations among variables within the two subsamples are presented in Table 2. The three proposed mediator variables – dangerousness, social distance, and concept breadth – significantly correlated with each other and with help-seeking attitudes in both subsamples, with the exception of the concept breadth and help-seeking attitude correlation among White Americans. The stigma scales were only modestly associated with the concept breadth measure (*rs* from 0.13 to 0.30), supporting the distinctness of these constructs. The relationships between gender and key variables were tested with independent sample *t*-tests on the combined sample. Men scored significantly lower on help-seeking attitudes than women, *t*(210) = −2.24, *p* = 0.03, 95% CI [−5.57, −0.36]. No other significant gender differences were obtained.

**Table 2:** Correlations among variables by group.

S. No.		1	2	3	4	5	6	7
1.	Age		0.28^**^	−0.01	0.15	0.21^*^	−0.07	0.03
2.	Education	0.03		0.37^***^	0.23^*^	0.21^*^	−0.09	0.02
3.	Income	−0.09	0.19		0.17	0.14	−0.12	0.12
4.	Dangerousness	0.07	−0.05	0.11		0.71^***^	−0.27^**^	−0.25^*^
5.	Social distance	0.22^*^	0.01	0.07	0.84^***^		−0.30^**^	−0.22^*^
6.	Concept breadth	−0.07	−0.04	0.05	−0.13	−0.24^*^		0.20^*^
7.	Help-seeking attitudes	−0.10	0.02	0.08	−0.49^***^	−0.49^***^	0.18	

*Spearman’s rho correlations are presented for associations involving education and income. Correlations above and below the diagonal correspond to the Asian American (n = 102) and White American (n = 110) subsamples, respectively*.

* *p* < 0.05;

** *p* < 0.01; and

*** *p* < 0.001.

### Mediation Model With Stigma as the Mediator

Table 3 shows that Asian Americans had less positive help-seeking attitudes than White Americans, *t*(210) = −2.42, *p* = 0.02, 95% CI [−5.80, −0.59], in support of *H*_1_. Similarly, Asian Americans had a significantly greater desire to maintain social distance from people with mental disorders than White Americans, *t*(210) = 3.76, *p* < 0.001, 95% CI [0.16, 0.52], supporting *H*_2_ and social distance negatively correlated with help-seeking, supporting *H*_3_, *r*(210) = −0.39, *p* < 0.001. All assumptions of mediation (Tabachnick and Fidell, 2007; Kane and Ashbaugh, 2017) were therefore met.

**Table 3:** Pearson correlations and independent sample *t*-tests comparing groups for stigma, concept breadth, and help-seeking attitudes.

S. No.	Measures	Correlations				*t*-tests	
		1	2	3	4	*t*	*p*
1.	Social distance		0.80^***^	−0.29^***^	−0.39^***^	3.76	< 0.001
2.	Dangerousness			−0.23^**^	−0.40^***^	3.28	0.001
3.	Concept breadth				0.21^**^	−2.14	0.03
4.	Help-seeking attitudes					−2.42	0.02

*For correlations, n = 210. The degree of freedom of the t-tests between subsamples on social distance, dangerousness, and help-seeking attitudes was 210, and for concept breadth was 195.46*. ^*^p < 0.05;

** *p* < 0.01; and

*** *p* < 0.001.

**Figure 1:**
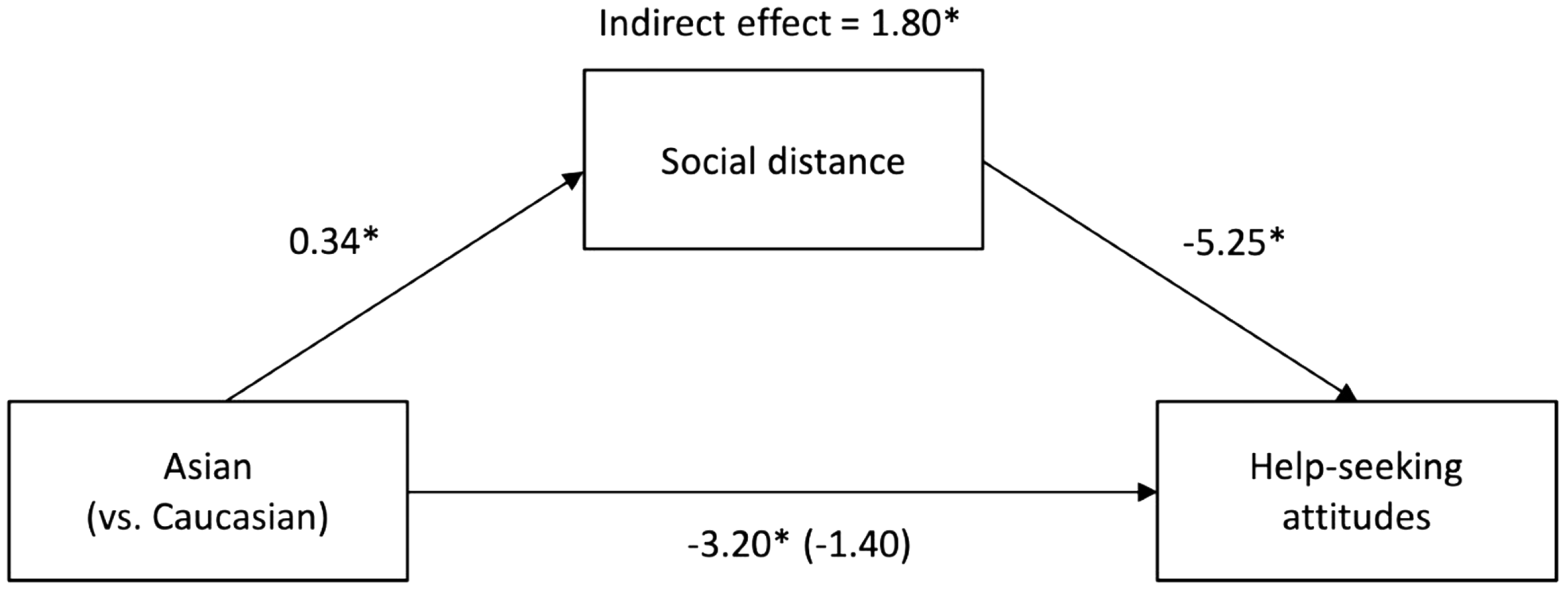
Mediation model with social distance as the mediator. Unstandardized regression coefficients for the relationship between group (Asian American: *n* = 102; White American: *n* = 110) and help-seeking attitudes as mediated by social distance. The unstandardized regression coefficient between group and help-seeking attitudes controlling for social distance is in parentheses. Indirect effect of social distance on the relationship from bootstrap analyses (*n* = 10,000). ^*^p < 0.05.

A mediation model using social distance as the mediator is illustrated in Figure 1. Asian Americans reported greater desire for social distance from people with mental disorder, *B* = −0.34, *SE* = 0.09, 95% CI [−0.52, −0.16], and a higher social distance was related to less positive help-seeking attitudes, *B* = −5.25, *SE* = 0.94, 95% CI [−7.10, −3.41]. The analysis revealed a significant indirect effect of group on help-seeking attitudes *via* social distance, consistent with *H*_4_, *B* = 1.80, *SE* = 0.53, 95% CI [0.90, 3.02]. Since there were group differences in age, income, and education, as well as the literature showing gender differences in help-seeking attitudes (e.g., Shea and Yeh, 2008), these demographic variables were included as covariates in the mediation model to test the robustness of the mediator. When controlling these covariates, the indirect effect of social distance remained significant, *B* = 2.33, *SE* = 0.66, 95% CI [1.21, 3.87]. The results for the model with dangerousness as the mediator were almost identical (e.g., significant indirect effect both without covariates, *B* = 1.65, *SE* = 0.58, 95% CI [0.69, 3.01], and with covariates, *B* = 1.91, *SE* = 0.71, 95% CI [0.71, 3.49]), reflecting the very strong correlation between social distance and dangerousness.

### Mediation Model With Concept Breadth as the Mediator

**Figure 2:**
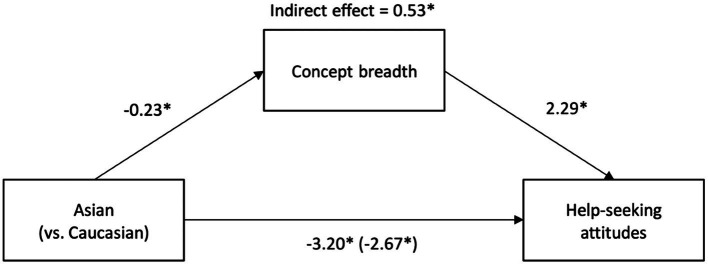
Mediation model with concept breadth as the mediator. Unstandardized regression coefficients for the relationship between group (Asian American: *n* = 102; White American: *n* = 110) and help-seeking attitudes as mediated by concept breadth. The unstandardized regression coefficient between group and help-seeking attitudes controlling for concept breadth is in parentheses. Indirect effect of concept breadth on the relationship from bootstrap analyses (*n* = 10,000). ^*^p < 0.05.

Table 3 shows Asian Americans had a narrower concept of mental disorder than White Americans, supporting *H*_5_. Consistent with *H*_6_, concept breadth and help-seeking attitudes were positively correlated. Figure 2 presents the mediation model. Asian Americans had a narrower concept of mental disorder than White Americans, *B* = 0.23, *SE* = 0.11, 95% CI [0.02, 0.44], a narrower concept was related to having less positive help-seeking attitudes, *B* = 2.29, *SE* = 0.84, 95% CI [0.63, 3.94], and there was a significant indirect effect of group on help-seeking attitudes *via* concept breadth, *B* = 0.53, *SE* = 0.32, 95% CI [0.07, 1.40], consistent with *H*_7_. This mediated effect remained significant when the covariates were added to the model, *B* = 0.48, *SE* = 0.33, 95% CI [0.02, 1.37].

## Discussion

The present study was the first to explore how the inclusiveness of the concept of mental disorder is associated with attitudes to mental health help-seeking and how differences in concept breadth may illuminate group differences in these attitudes. All study hypotheses received support. As we expected, Asian Americans and White Americans differed in their mean levels of help-seeking, attitudes, stigma, and concept breadth (*H*_1_, *H*_2_, and *H*_5_); stigma and concept breadth each predicted help-seeking attitudes (*H*_3_ and *H*_6_); and stigma and concept breadth each partially mediated the group difference in help-seeking attitudes (*H*_4_ and *H*_7_).

Several results replicate the established findings of the previous work. The higher average level of personal stigma in the Asian American group obtained here with both the perceived dangerousness and social distance measures of stigma, and the less positive attitudes to mental health help-seeking among Asian Americans, both accord with large bodies of the previous research (U.S. Department of Health and Human Services, 2001; Masuda et al., 2009; Yamawaki, 2010; Masuda and Boone, 2011). The finding that differences in stigma mediate the relationship between cultural group and help-seeking attitudes replicates the important work of Loya et al. (2010). It also extends that work by sampling the general population rather than college students and by sampling Asian Americans in general rather than South Asian Americans in particular. As others have argued, the higher average level of stigma in the Asian American subsample may reflect a greater concern with the shame that mental illness might bring to family or community than other Americans. The greater shame surrounding mental disorder may discourage help-seeking.

The hypotheses concerning concept breadth were equally well supported as those concerning stigma, but the former were more novel, as the relationship between stigma and help-seeking has been well established in the previous studies. Asian Americans held less expansive concepts of disorder, expansive concepts were associated with more positive attitudes to help-seeking, and holding expansive concepts mediated the group difference in help-seeking attitudes. As with stigma, although the two groups had some demographic differences, all findings were robust when these differences were controlled. Although cultural differences in conceptualizations of mental disorders have often been proposed (e.g., Ng, 1997), few quantitative studies have investigated them in the context of cultural differences in mental health attitudes. Very few have addressed such differences through the lens of concept breadth: The range of phenomena perceived to be mental disorders. Prior work on this topic has exclusively examined differences between national-level cultures (Giosan et al., 2001) or variations within a group as a function of acculturation (Glovsky and Haslam, 2003). Hence, the present study’s examination of ethnic or racial differences within a country and linkages of these differences to mental health-related phenomena is innovative.

Why Asian Americans might have narrower concepts of mental disorder than White Americans is uncertain. One possibility is that Asian Americans may tend to have a more interdependent cultural orientation than other Americans, placing less emphasis on individuals’ emotional distress unless it disrupts social harmony. Consequently, they may be less apt to recognize disorders that primarily reflect personal distress or dysfunction rather than social disruption. Alternatively, the tendency for people of Asian ethnicities to experience psychological distress in a more somatic fashion than other groups (Ryder et al., 2008; Maffini and Wong, 2014) may be associated with an inclination to view a narrower range of experiences as mental disorders specifically. It is also possible that there are varying thresholds in judging if someone has a mental disorder. For instance, White Americans caregivers were more likely to rate the mental health of their adolescents as only fair or poor, compared to their African or Latino counterparts (Roberts et al., 2005). However, it is also possible that other as-yet-unknown cultural factors (e.g., narrower concepts of abnormality in Asian languages, greater social conservatism among Asian American populations, or lesser familiarity with Western psychiatry, which has undergone substantial diagnostic inflation in recent decades) may underpin the group difference in concept breadth.

The finding that having more restricted concepts of mental disorder was associated with less positive attitudes to help-seeking is a novel one. This is consistent with the theoretical speculation that holding narrower concepts of disorder would translate into a lower likelihood of acknowledging a problem as a mental disorder, the first stage of help-seeking (Featherstone and Broadhurst, 2003). However, it is also possible that a third factor accounts for narrow concepts and less positive attitudes. For example, people who hold negative views of the mental health professions or believe that current concepts of mental disorder pathologize or excuse socially deviant behavior might hold narrow concepts of disorder and negative views of psychological services. Future research is warranted to clarify the basis of this concept-attitude link. In particular, longitudinal research is required to test whether the mediating role of concept breadth in group differences in help-seeking attitudes reflects a causal influence of concept breadth. Future research might also explore whether there may be costs, as well as benefits, of holding more inclusive concepts of mental disorder, such as a greater sense of personal vulnerability.

Our unique finding that differences in concept breadth explain group differences in help-seeking attitudes may have practical implications. If broader concepts of disorder are associated with greater treatment utilization *via* more positive help-seeking attitudes, then public mental health campaigns might aim to broaden these concepts rather than focusing exclusively on destigmatizing disorders. Despite continuing efforts to reduce stigma, a 40-year cross-temporal meta-analysis suggested that help-seeking attitudes have been increasingly negative (Mackenzie et al., 2014), pointing to the need to identify new targets for intervention. To this end, it is particularly important to acknowledge the weak but present negative relationship between concept breadth and stigma (mean *r* = −0.24), indicating that they represent two related but distinct constructs. The relationship between concept breadth and stigma might reflect greater normalization of mental disorder among people who hold expansive concepts of it but it also might be explained in part by defensive projection. Someone who holds a negative view of mental disorders and has some symptoms of mental disorder may be more likely not to define similar symptoms or experiences as mental disorders in order to protect themselves from being part of the stigmatized group (Zoubaa et al., 2020). This link between stigma and concept breadth implies that targeted campaigns would help to broaden the public’s understanding of the range of problems that would benefit from mental health interventions, and hence, might usefully complement existing stigma reduction programs.

Building awareness of group differences in the breadth of concepts of mental disorder may also have benefits for clinicians. Although cultural differences are acknowledged in the *DSM-5* (Regier et al., 2013), practitioners may be unaware that some potential and existing patients may have understandings of what counts as a mental disorder that differ widely from one another based on cultural background. Patients and family members from groups that hold relatively restrictive concepts of disorder may be reluctant to seek help, ambivalent about help once it has been sought, and likely to interpret experiences and behaviors that mental health professionals view as pathological in other, non-psychiatric ways. More generally, mental health professionals may lack awareness that public understandings of mental disorder may be substantially narrower than their own. Tikkinen et al. (2019), for example, recently showed that psychiatrists define a broader set of mental health conditions to be diseases than non-psychiatrist physicians, who in turn had more inclusive concepts than laypeople. Greater awareness of such discrepancies and their clinical implications among professionals might improve treatment engagement and effectiveness.

Several limitations of this research should be acknowledged. First, the focus on groups within a single country does not allow confident conclusions about ethnic or racial differences in concepts of mental disorder in other countries, or inferences about dominant concepts of disorder in Asian countries. It will be important for future research to replicate group differences in other national contexts and to explore concept breadth in other cultural and linguistic contexts. As Asian Americans are a diverse population, containing varied ethnicities with very different histories of immigration, it will also be important to explore variations between individuals of different ethnicities, immigration statuses, and levels of acculturation. The modest size of the present study’s sample did not allow such variations to be examined, and the study may therefore obscure significant variability within the Asian American population. Second, our focus on help-seeking attitudes rather than behaviors as outcomes makes it risky to conclude that differences in concept breadth translate into actual change in treatment uptake. Finally, our cross-sectional design does not allow inferences about the direction of associations. Concept breadth may merely covary with attitudes to seeking help for mental health problems rather than influence them causally, and attitudes to help-seeking might reciprocally affect concepts of disorder. Experimental or longitudinal research might help to clarify the nature of these relationships.

## Conclusion

The present research indicates that the breadth of people’s concepts of mental disorder plays a previously unrecognized role both in help-seeking attitudes and group differences in those attitudes. Holding an inclusive concept of disorder, and holding a less stigmatizing view of disorder, may therefore both promote help-seeking. These findings significantly extend previous research on differences between Asian and White Americans in help-seeking (Loya et al., 2010), and point to a potentially important new factor that efforts to boost help-seeking might consider, especially if they are culturally informed. Future research should aim to assess the mechanisms and implications of mental disorder concept breadth and explore its cross-cultural applicability.

## Data Availability Statement

The raw data supporting the conclusions of this article will be made available by the authors, without undue reservation.

## Ethics Statement

The studies involving human participants were reviewed and approved by the University of Melbourne Human Research Ethics Committee. The patients/participants provided their written informed consent to participate in this study.

## Author Contributions

JT and NH designed the research and drafted the manuscript. JT carried out the data collection and analysis under NH’s supervision. All authors contributed to the article and approved the submitted version.

## Conflict of Interest

The authors declare that the research was conducted in the absence of any commercial or financial relationships that could be construed as a potential conflict of interest.

## Publisher’s Note

All claims expressed in this article are solely those of the authors and do not necessarily represent those of their affiliated organizations, or those of the publisher, the editors and the reviewers. Any product that may be evaluated in this article, or claim that may be made by its manufacturer, is not guaranteed or endorsed by the publisher.
